# Green Plants in the Red: A Baseline Global Assessment for the IUCN Sampled Red List Index for Plants

**DOI:** 10.1371/journal.pone.0135152

**Published:** 2015-08-07

**Authors:** Neil A. Brummitt, Steven P. Bachman, Janine Griffiths-Lee, Maiko Lutz, Justin F. Moat, Aljos Farjon, John S. Donaldson, Craig Hilton-Taylor, Thomas R. Meagher, Sara Albuquerque, Elina Aletrari, A. Kei Andrews, Guy Atchison, Elisabeth Baloch, Barbara Barlozzini, Alice Brunazzi, Julia Carretero, Marco Celesti, Helen Chadburn, Eduardo Cianfoni, Chris Cockel, Vanessa Coldwell, Benedetta Concetti, Sara Contu, Vicki Crook, Philippa Dyson, Lauren Gardiner, Nadia Ghanim, Hannah Greene, Alice Groom, Ruth Harker, Della Hopkins, Sonia Khela, Poppy Lakeman-Fraser, Heather Lindon, Helen Lockwood, Christine Loftus, Debora Lombrici, Lucia Lopez-Poveda, James Lyon, Patricia Malcolm-Tompkins, Kirsty McGregor, Laura Moreno, Linda Murray, Keara Nazar, Emily Power, Mireya Quiton Tuijtelaars, Ruth Salter, Robert Segrott, Hannah Thacker, Leighton J. Thomas, Sarah Tingvoll, Gemma Watkinson, Katerina Wojtaszekova, Eimear M. Nic Lughadha

**Affiliations:** 1 Natural History Museum, Cromwell Road, South Kensington, London, United Kingdom; 2 Royal Botanic Gardens, Kew, Richmond, Surrey, United Kingdom; 3 School of Geography, University of Nottingham, Nottingham, United Kingdom; 4 South African National Biodiversity Institute, KRC, Private Bag X7, Claremont, South Africa; 5 IUCN Red List Unit, Sheraton House, Castle Park, Cambridge, United Kingdom; 6 School of Biology, Dyers Brae, University of St Andrews, St Andrews, Fife, United Kingdom; 7 King's College London, Strand, London, United Kingdom; Instituto de Pesquisas Ecológicas, BRAZIL

## Abstract

Plants provide fundamental support systems for life on Earth and are the basis for all terrestrial ecosystems; a decline in plant diversity will be detrimental to all other groups of organisms including humans. Decline in plant diversity has been hard to quantify, due to the huge numbers of known and yet to be discovered species and the lack of an adequate baseline assessment of extinction risk against which to track changes. The biodiversity of many remote parts of the world remains poorly known, and the rate of new assessments of extinction risk for individual plant species approximates the rate at which new plant species are described. Thus the question ‘How threatened are plants?’ is still very difficult to answer accurately. While completing assessments for each species of plant remains a distant prospect, by assessing a randomly selected sample of species the Sampled Red List Index for Plants gives, for the first time, an accurate view of how threatened plants are across the world. It represents the first key phase of ongoing efforts to monitor the status of the world’s plants. More than 20% of plant species assessed are threatened with extinction, and the habitat with the most threatened species is overwhelmingly tropical rain forest, where the greatest threat to plants is anthropogenic habitat conversion, for arable and livestock agriculture, and harvesting of natural resources. Gymnosperms (e.g. conifers and cycads) are the most threatened group, while a third of plant species included in this study have yet to receive an assessment or are so poorly known that we cannot yet ascertain whether they are threatened or not. This study provides a baseline assessment from which trends in the status of plant biodiversity can be measured and periodically reassessed.

## Introduction

### Responding to global biodiversity targets

At the tenth conference of the parties to the Convention on Biological Diversity (CBD) it was widely agreed that the 2010 Biodiversity Target of a significant reduction in the rate of biodiversity loss [1–2] had not been met [3]. The Parties to the Convention responded to this by proposing a follow-on to the 2010 International Year of Biodiversity, declaring a Decade on Biodiversity and setting 20 new ‘Aichi’ biodiversity targets to be met by the year 2020 (Decision X/2) [4]. The IUCN Red List of Threatened Species (hereafter referred to as the IUCN Red List) is widely recognised as the most authoritative information source on the extinction risk of species [5–7] and the IUCN Red List Index [8–9], in which genuine changes in IUCN Red List status over time are used to generate an overall index of change, is one of four indicators to measure progress towards Target 12 of the new CBD Strategic Plan (SBSTTA Recommendation XV/1; www.bipindicators.net/globalindicators): that ‘*the extinction of known threatened species has been prevented and their conservation status*, *particularly of those most in decline*, *has been improved and sustained*’ by 2020.

The IUCN Red List Index was first calculated for the world’s birds [3] and has now been applied to other groups of species (e.g. mammals [10], amphibians [11], warm water reef-building corals [12] and freshwater crayfish [13]). The IUCN Sampled Red List Index (SRLI) [14] is an extension of the IUCN Red List Index to larger and less well known groups of organisms that have not been comprehensively assessed; it complements the results for comprehensively assessed groups by representing a broader swathe of global biodiversity but without requiring full assessments for all species in a group. The SRLI contributes to the ‘Barometer of Life’ [15]: it captures the status of life on earth at a particular moment, and how this changes over time. Baseline values for a Sampled Red List Index have already been calculated for several large but under-assessed groups such as freshwater crabs [16], dragonflies and damselflies [17] and reptiles [18], and work is progressing for butterflies and moths [19] and other groups, providing a better representation of the extinction risk of biodiversity as a whole; from such a baseline, trends can be estimated once these samples have been re-assessed.

Plants are an essential component of biodiversity for which, until now, there has been no global assessment of overall extinction risk. The SRLI for Plants addresses this important gap. It also responds to other global policy mechanisms such as the Global Strategy for Plant Conservation (GSPC) [20], of which Target 2 is a conservation assessment of all plant species, also to be delivered by 2020. Until such time as an assessment of every plant species can be achieved the SRLI for Plants serves as a proxy for overall plant extinction risk and is specifically cited as milestone (d) under GSPC Target 2 (UNEP) [21].

### IUCN Red List assessment and extinction risk of plants

Plants provide fundamental support systems for life on Earth and are the basis for all terrestrial ecosystems, and a decline in plant diversity will be detrimental to all other groups of organisms including humans [22–23]. Decline in plant diversity has been hard to quantify, due to the huge numbers of known [24] and unknown [25] species and the lack of an adequate baseline on the extinction risk of species against which to track changes. The IUCN Red List remains the most widely-used and authoritative source of information on threatened plant species worldwide, but the rate at which plant species are described as new to science (more than 2,000 each year–average for last ten years: 2135 [http://www.ipni.org/stats.html]) is nearly equivalent to the number of new IUCN Red List assessments of plants being published each year (approximately 1500 per year since 2001, although the rate is increasing). Significant progress on IUCN Red List assessments has been made for plants at the national and regional level [26–27] and, despite some difficulties with applying IUCN Red List Criteria to global assessments of plants [28–29], to date assessments for 19,738 plants have been published on the IUCN Red List [30].

However, there remains a great need for additional assessments of plant extinction risk [31], and using those plant assessments currently available on the IUCN Red List as a resource to understand the broader status and trends in plant extinction risk is problematic. Firstly, a large proportion of plants already assessed on the IUCN Red List are historic assessments, largely derived from a landmark publication on the extinction risk of tree species [32], which are already in need of re-assessment [33]. Secondly, the current sample of the world’s plants on the IUCN Red List gives a biased view of overall extinction risk, mainly because over many years most effort has been focussed on assessing those species that scientists expected to be threatened, due both to the limited resources available for assessments and to a natural tendency to select species considered to be most in need of assessment. The IUCN Red List as it stands thus overestimates the overall proportion of plant species threatened, a bias that is unlikely to diminish until assessments of the majority of plant species are added to the IUCN Red List [34]. The present proportion of plant species listed on the IUCN Red List [30] as threatened is 54%, although this is not reported because IUCN recognise that there is insufficient coverage of assessments across all plant groups to report an accurate percentage of threatened species for plants as a whole. Using a sampled approach [14, 28], the SRLI for Plants tackles this problem by selecting a sample of plant species at random, so that it is representative of plants as a whole. The assessment of several thousand plant species presented here sets the first baseline of global plant extinction risk against which future changes can be measured.

## Methods

### Species assessed and species selection

From the almost 380,000 known plant species, a random sample of 7,000 species was selected for the SRLI for Plants [28]. Species from five major groups of plants are included (total species numbers for each group follow in square brackets): bryophytes (mosses, liverworts and hornworts) [34,556 species]; pteridophytes (true ferns, lycophytes and allied species) [12,838 species]; gymnosperms (conifers, cycads, gnetophytes and *Ginkgo biloba*) [1,032 species]; monocotyledons (one of the major groups of flowering plants, which includes orchids, bulbous plants, palm trees, aroids and the grass and sedge families) [71,445 species]; and the legume family (peas and beans) [19,874 species]. The legume family was selected as a surrogate for the much larger eudicot clade, for which no authoritative global checklist was available at the time the sample was taken [2008]. The legume family is a large but relatively well-studied family [35] for which a global checklist is available (www.ILDIS.org), and that has been shown to be the angiosperm family which best mirrors overall patterns of global angiosperm diversity [36].

Data sources sampled for each group were: bryophytes: Index of Mosses (http://www.mobot.org/MOBOT/tropicos/most/iom.shtml); pteridophytes: Checklist of World Ferns (http://homepages.caverock.net.nz/~bj/fern/list.htm); gymnosperms: Handbook of the World’s Conifers [37], the Cycad Pages (http://plantnet.rbgsyd.nsw.gov.au/PlantNet/cycad/) and the World Checklist of Selected Plant Families (http://apps.kew.org/wcsp/), for *Ephedra* and *Gnetum*; monocots: the Monocot Checklist now incorporated into the World Checklist of Selected Plant Families (http://apps.kew.org/wcsp/); legumes: the International Legume Database and Information Service–ILDIS (http://www.ildis.org/). Taxonomic consistency of all samples taken was subsequently improved by contributions from numerous taxonomic and regional experts, from consulting numerous national checklists, Flora accounts and taxonomic treatments, and through peer-review of the assessments as they were completed. If, at the time of assessment, names taken from the original sample were revealed to be homotypic synonyms of currently accepted taxa (75 cases in the sample of monocot species) and the conservation status was considered to be unaffected, the synonym was replaced in our sample by the accepted name. Heterotypic synonyms (97 monocot species) were also replaced by the accepted name apart from a small number where defaulting to the accepted name was considered likely to produce a different assessment of extinction risk, in which case names were replaced in our sample with other species names selected at random from the relevant list, lest defaulting to the accepted name should introduce a bias towards widespread species. Heterotypic synonyms usually post-date the accepted name to which they are referable, and these more recently described taxa tend to have smaller ranges [38–39].

In order to establish a reasonable sample size to ensure credible taxonomic and geographical breadth in the SRLI samples, an analysis of sample coverage was done using the monocots. The Monocot Checklist (~70,000 species) was successively re-sampled randomly without replacement in increments of 250, and at each increment the Shannon-Weiner and Simpson’s indices of diversity [40] were calculated up to a final sample size of 3,000 species; 1,000 iterations per sample size increment were performed (see Supporting Information). Diversity indices measure the number of types in a set of data and how the units are distributed among these types; in this case, units were the number of species and the types are either taxonomic families or geographic region, and this was calculated against the known diversity statistics for the Monocot Checklist as a whole. The objective was to determine the sample size at which a random sample of species yielded a measure of diversity equivalent to either index of diversity for the whole group, and to determine upper and lower bounds of diversity on samples of varying size.

For the major taxonomic groups of plants assessed–except one– 1,500 species were selected randomly without replacement for assessment; the exception is the gymnosperms, of which there are fewer than 1,500 species in the world, so all gymnosperm species are included here, combining assessments by the IUCN SSC Conifer Specialist Group and the IUCN SSC Cycad Specialist Group together with assessments for remaining gymnosperm species carried out by the SRLI for Plants project team.

The sample size of 1,500 species used for the SRLI for Plants is also sufficient to accommodate in-built redundancy in assessments [14] due to taxonomic change/uncertainty or inadequate data being available for some species. Based on simulations using the Red List assessments for all bird species, and confirmed using the Red List assessments for all mammals and all amphibians, it has been determined that to calculate the SRLI with 95% confidence, a threshold number of at least 900 assessments of each sample of 1,500 species (60%) need to be ‘data sufficient’ and rated as Least Concern (LC), Near Threatened (NT), Vulnerable (VU), Endangered (EN) or Critically Endangered (CR), and not as Data Deficient (DD) [14]. This approach recommended by Baillie et al. [14] and followed here therefore implicitly assumes that the variation in extinction risk for plants will be equivalent to that shown by birds, mammals and amphibians. The threshold of 900 species assessed was met for each of pteridophytes, gymnosperms, monocots and legumes; only preliminary results are so far available for the bryophytes, and results from this group are not currently included in the SRLI for Plants or presented here, except for the preliminary results in Table 1. Apart from Table 1, all other results presented include samples for pteridophytes, monocots and legumes together with the complete assessment of gymnosperms, a combined sample size of 3990 non-DD species assessed. The value of the SRLI for Plants was calculated following the modified formula of Butchart et al. [41].

**Table 1:** Numbers of species from the combined SRLI for Plants sample of gymnosperms, monocots, legumes and pteridophytes by IUCN Red List Category for each group of plants.

	Monocotyledons	Legumes	Gymnosperms	Pteridophytes	Bryophytes (preliminary results only)	Total	Total excluding bryophytes
**CR**	32	14	80	18	40	**184**	**144**
**EN**	55	51	163	46	36	**351**	**315**
**VU**	71	39	156	91	22	**379**	**357**
**Threatened**	**158**	**104**	**399**	**155**	**98**	**914**	**816**
**lower estimate % threatened**	15.46	10.53	39.58	15.95	15.51	**19.77**	**20.45**
**best estimate % threatened**	**17.59**	**11.43**	**40.38**	**16.01**	**19.60**	**21.44**	**21.68**
**upper estimate % threatened**	27.59	18.42	41.57	16.36	36.39	**27.52**	**26.12**
**NT**	67	74	167	55	43	**406**	**363**
**LC**	677	732	418	758	359	**2944**	**2585**
**DD**	124	78	20	4	132	**358**	**226**
**Subtotal**	1026	988	1004	972	632	**4626**	**3990**
**NE**	478	512	24	528	868	**2410**	** **
**Total**	**1500**	**1500**	**1028**	**1500**	**1500**	** **	** **

### Assessment methodology

The extinction risk of each species was assessed using the IUCN Red List Categories and Criteria version 3.1, during the period 2007–2015. [42]. Since the majority of plant species lack any documentation on population sizes or dynamics, for most species the most comprehensive, easily accessible and reliable information on which to base a conservation assessment is the known distribution of that species, utilising Criterion B, although all criteria were considered for each assessment [43]. The best source of this distribution information is the collection of plant specimens held in the world’s herbaria [28]. Herbarium specimens provide verifiable records indicating the existence of a species at a given time and place. Historical specimens may play a role in assessments as they can indicate that a decline has occurred, if contemporary surveys reveal that the species is no longer extant at a locality where a historical collection was made. For the purpose of range based measures using Criterion B however, only those specimens considered representative of the present day range should be used and automated tools have been developed to facilitate incorporation of these specimen records into IUCN Red List assessments [44–45]. The herbarium collections at the Royal Botanic Gardens, Kew contain some seven million plant and fungus specimens, and the Natural History Museum, London contains six million plant specimens from all over the world. These were systematically searched for all available specimen information for a given species, and all collections databased and geo-referenced, together with other specimen data available online, such as institutional data portals, e.g. TROPICOS (http://www.mobot.org/MOBOT/tropicos/) or the Global Biodiversity Information Facility (GBIF; www.gbif.org), used with due caution and carefully reviewed and edited to remove duplicate records and erroneous geo-references and where necessary geo-referenced or re-geo-referenced by the project team. These specimen data were combined with information about the species from scientific literature, analysis of the species range using Geographical Information Systems (GIS) together with widely available global datasets such as the Human Footprint Index [46], the World Database on Protected Areas [47] and the Gridded Population of the World [48] and freely available satellite imagery such as in Google Earth, and the expert opinion of scientists who study that species or the area of the world where it is found, to assess the extinction risk for that species and assign an IUCN Red List Category [28].

Species are assessed for the IUCN Red List using any or all of five quantitative criteria A–E [42]. Spatial parameters within the IUCN criteria lend themselves to calculation within a GIS; by collating all known specimens for a species, filtering which of those accurately represent the present distribution, and plotting them on a map it is possible to determine area-based measures such as extent of occurrence (EOO) and area of occupancy (AOO) [28,43,45,49], even for those known from only a handful of specimens [50], and evaluate these metrics against the relevant thresholds for threatened categories [42]. This approach assumes that point distributions adequately capture the extent and occupancy of the species range. For EOO, there is a simple geometric constraint of at least three unique localities required to build the minimum convex polygon (MCP), the metric used to determine EOO [44–45], although sampling intensity should be sufficient such that those three points represent the extent of the species. The estimation of AOO, in particular, is affected by sampling intensity so that it was only used when ‘genuine’ absence in 2 km x 2 km cells could be inferred, for example a species with a restricted, isolated range on a mountain top that had been well sampled. In extreme cases, even when only a single type specimen is known, it still may be possible to use the AOO measure, depending on the contextual data available [43]. This rapid, automated approach produces a preliminary assessment because further subcriteria must be met to fully justify an IUCN Red List rating [28]. All preliminary assessments were evaluated to determine whether at least two out of three sub-criteria under Criterion B are met, such as a continuing decline in or severe fragmentation of a species’ range, so that the assessment can be described as ‘full’, subject to verification by an independent evaluator. If the additional sub-criteria were not met, the species were either rated as Least Concern, or Near Threatened if the sub-criteria were partially met and were close to the VU thresholds, or DD if there was insufficient information to make a judgement.

Species newly assessed for the SRLI for Plants have associated point data that have either been submitted to, or are in the process of being submitted to IUCN, to verify the area-based calculations and to fulfil the minimum data requirements for IUCN Red List assessments. Species assessed from 2007 onwards by other Red List Authorities or during current assessment programmes were generally not re-assessed, but if a species had previously been assessed and was already listed on the IUCN Red List under older criteria, it was re-assessed under the current IUCN Categories and Criteria (v. 3.1) [42], with a new rating [IUCN Red List Category] as necessary or not. Any species that had previously been assessed under a different (non-IUCN) classification system, such as NatureServe conservation status ranks [51], were re-assessed using IUCN Categories and Criteria (v. 3.1) [42].

Assessments were made available online for peer-review (www.threatenedplants.myspecies.info) and/or sent directly to taxonomic or regional experts or IUCN Red List authorities, and expert workshops held to evaluate individual species ratings. All species assessments were referred to the relevant IUCN Red List Authorities (RLAs) for review as required by the IUCN submissions process. For species not covered by an RLA, these were submitted directly to the IUCN Red List Unit to review or to find alternative experts to conduct the review. Reviewed assessments were forwarded to the IUCN Red List Unit for final checking and publication on the IUCN Red List. By the publication of the 2014.3 IUCN Red List update [30], assessments of 1,009 gymnosperms, 1,005 monocots and 967 legumes have been published, with 25 draft cycad assessments plus 2 in need of updating and 25 further monocot assessments still under review; 972 ferns have been submitted for publication. The remaining species in the sample have yet to receive a full assessment and are listed as Not Evaluated (NE). It is important to emphasize here that the majority of Not Evaluated species have been investigated but put to one side for now as there appeared to be little information on which to base an assessment. These have not been formally rated as Data Deficient, although there is an assumption that most of these will turn out not to be threatened, but these species await further investigation when time allows since NE and DD species do not contribute to the overall SRLI for Plants value. As further data and resources become available these assessments will be carried out and backcast to synchronise with the other SRLI Phase 1 assessments [29].

### Proportions of species threatened

The proportion of threatened species for each group was calculated as [Number of threatened / (Total—DD)], where 'threatened' is the number of species assessed as either Vulnerable (VU), Endangered (EN), or Critically Endangered (CR), 'Total' is the total number of species assessed (i.e. excluding species that are Not Evaluated, NE) and DD is the number of species assessed as Data Deficient [10,52]. This gives a ‘best estimate’ of the percentage of species threatened under IUCN Red List Criteria that effectively assumes that DD species would be assessed with the same proportion threatened–and the same proportions in each of the threatened categories–as the ‘data sufficient’ species, if there was enough information known about the DD species in order to assess them; the lower estimate is given by [Number of threatened / Total]–thus assuming that no DD species will turn out to be threatened–and the higher estimate by [(Number of threatened + DD) / Total]–thus assuming that all DD species will be assessed as threatened [19,52]. Since many DD species are poorly known as a result of having very small ranges and/or population sizes compared with most data sufficient species, they might be considered more likely to be threatened under IUCN Red List Criteria [53]. However it is not possible to know this with certainty as, by definition, those DD species do not have enough information for them to be assessed.

As the number of species sampled varies between countries, some countries have a very high variance in estimates of the proportion of species threatened, resulting in misleadingly high or low proportions of threatened species for some countries, simply due to low sample size. To account for this, a re-sampling exercise was conducted to establish a threshold number of species sampled per country below which the proportion of species assessed as threatened is likely to be misleading (i.e. where sampling is too low—see Supporting Information and ‘SRLI value and geographic patterns‘ in Results). For sample sizes of one to 250 species, the standard error (SE) of sampled species rated as threatened was determined for 5,000 independent trials and a threshold determined below which the SE is asymptotic (this value is an effective balance between proportions of species that have little support in countries where there are few sampled species assessed, and representing sufficient countries to show global patterns of threatened plant species). This was at a value of less than 0.1 SE, and so proportions of species assessed as threatened or as Data Deficient are not reported for countries with 16 species or fewer.

## Results

### Diversity statistics

Diversity statistics for random samples of monocot species ((Shannon-Weiner Index and Simpson’s Index) stabilise over a narrow range as sample sizes increase beyond 1,250–1,500 species for both species per family (S1 Fig) and species per geographic region (S2 Fig). Random samples at or above these values yield diversity statistics that fall within a narrow range of variability. Thus, we conclude that randomly-selected samples of 1,500 ensure a robust coverage of the whole of the monocotyledons both taxonomically and geographically. This sample size is corroborated by modelling done by Baillie et al. (14) to test the repeatability of the SRLI for different sample sizes. For the following results presented by region and by habitat, and the figures summarising threats to plant species and proportions of threatened species by country, all four plant groups assessed have been analysed together (3990 non-DD species in total) rather than separately, so that the set of species within each region, habitat or country is large enough for there to be confidence in these results.

### Proportion of threatened plant species

Overall, more than one in five (almost 22% using the ‘best’ estimate of the proportion of threatened species) of plant species assessed are threatened with extinction, while close to one in three (30%) of all plant species assessed of elevated conservation concern, being either threatened or Near Threatened (that is, they are close to meeting the criteria for one of the threatened categories and are likely to become threatened if no steps are taken to halt their decline). The proportion of species threatened varies between the four groups assessed to date (Table 1), with only 11% of species estimated to be threatened for legumes, 16% for pteridophytes, 18% for monocots and 40% for gymnosperms; however, for each of these major groups many more species (between 7% and 17%) are classified as Near Threatened, and up to 50% of species are either threatened or Near Threatened in the case of gymnosperms (Table 1). A very high proportion of species, just over a third (34%), remains classified as either Data Deficient (6% of species assessed) or Not Evaluated (28% of the original sample), meaning not enough is currently known about them to complete an IUCN Red List assessment (DD) or that an assessment cannot yet be completed (NE). Almost two-thirds (65%) of plant species assessed for this study have been assessed as Least Concern and have a low probability of extinction (Table 1).

**Fig 1:**
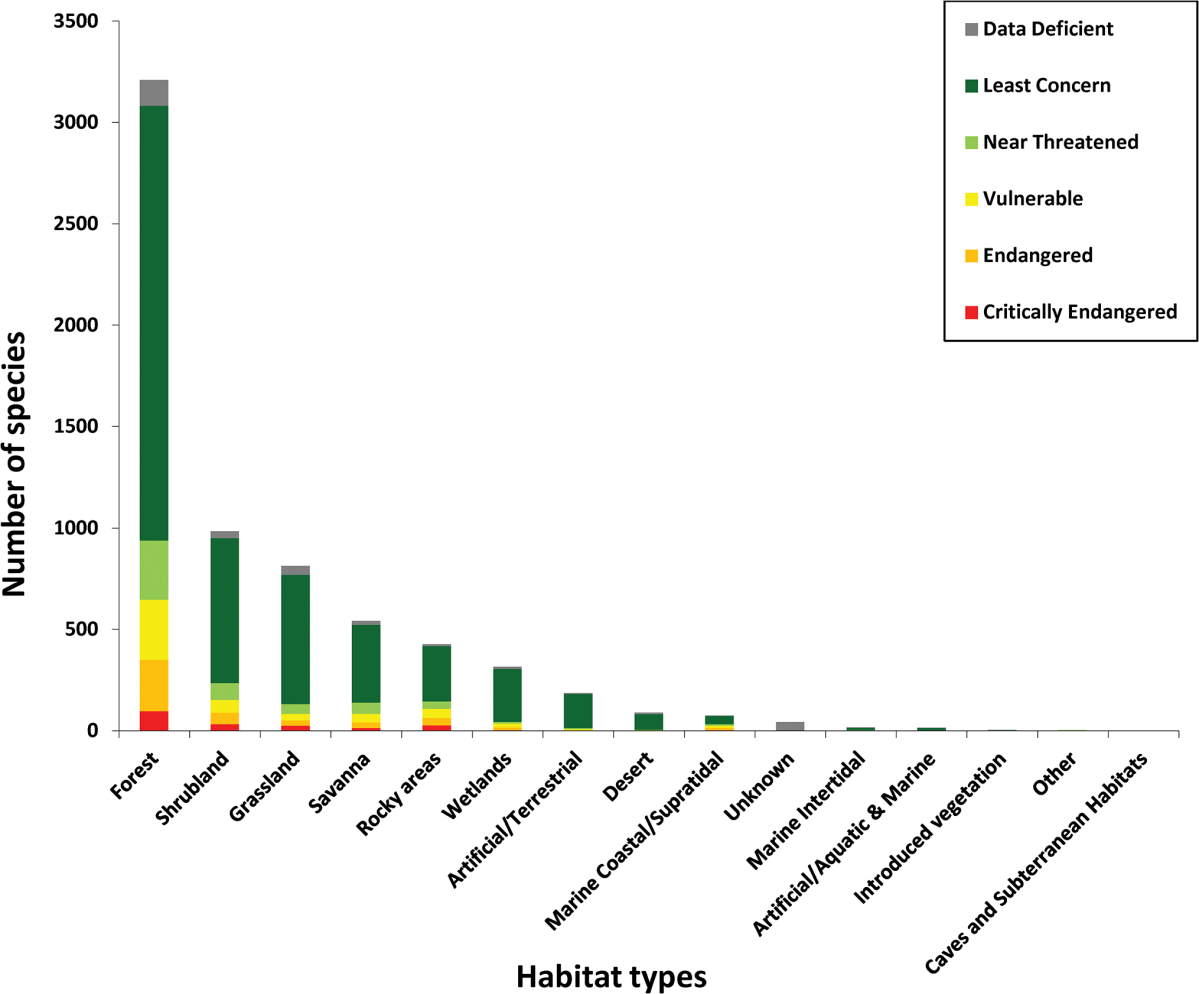
Numbers of species from the combined SRLI for Plants sample of gymnosperms, monocots, legumes and pteridophytes in each IUCN Red List Category by habitat.

The species included in this study are spread across major plant groups, habitats and regions of the world; because of the random nature of the sample, the numbers of species are in proportion to the plant diversity within each habitat or region. The species assessed can be divided into the habitats (Fig 1) and realms (Table 2) of the world in which they are known to occur. Discounting Antarctica and surrounding sub-Antarctic islands, which only have five species assessed for the SRLI for Plants, the region of the world with the greatest threats affecting its plant diversity is the Neotropics, with 23% of assessed species threatened, with tropical Africa (22%) and tropical Asia (21%) only just behind. Australasia, with a very large number and high proportion of endemic species, also has a high proportion of assessed species threatened (18%), while the islands of Oceania have 16% threatened. Lower proportions of the flora are threatened in temperate Eurasia (12%) or North America (10%) in this study (Table 2). The habitat with the greatest number of threatened species is clearly forest, with 79% of threatened species found in this habitat, followed by 19% of threatened species in shrubland, 13% in rocky outcrops, 10% in savanna and also 10% in grassland (Fig 1; percentages do not sum to 100% as some species occur in more than one habitat). Habitats with the greatest proportions of threatened species are all areas undergoing rapid conversion to agriculture. Habitats that contain the smallest proportions of threatened species, such as deserts and wetlands, are, perhaps unsurprisingly, areas least suitable for conversion to agriculture.

**Table 2:** Numbers of species from the combined SRLI for Plants sample of gymnosperms, monocots, legumes and pteridophytes in each IUCN Red List Category by realm.

	Afrotropical	Antarctic	Australasian	Indomalayan	Nearctic	Neotropical	Oceanian	Palearctic
**CR**	33	1	11	21	8	59	2	10
**EN**	50	0	44	65	19	108	6	29
**VU**	65	0	68	77	13	109	3	41
**NT**	57	0	82	71	34	95	2	46
**LC**	459	4	474	534	324	854	55	530
**DD**	49	0	26	54	1	55	1	48
**Total # species**	**713**	**5**	**705**	**822**	**399**	**1280**	**69**	**704**
**Total threatened**	**148**	**1**	**123**	**163**	**40**	**276**	**11**	**80**
**Lower estimate % threatened**	**20.76**	**20.00**	**17.45**	**19.83**	**10.03**	**21.56**	**15.94**	**11.36**
**Best estimate % threatened**	**22.29**	**20.00**	**18.11**	**21.22**	**10.05**	**22.53**	**16.18**	**12.20**
**Upper estimate % threatened**	**27.63**	**20.00**	**21.13**	**26.40**	**10.28**	**25.86**	**17.39**	**18.18**

### The impact of humans on plant diversity

**Fig 2:**
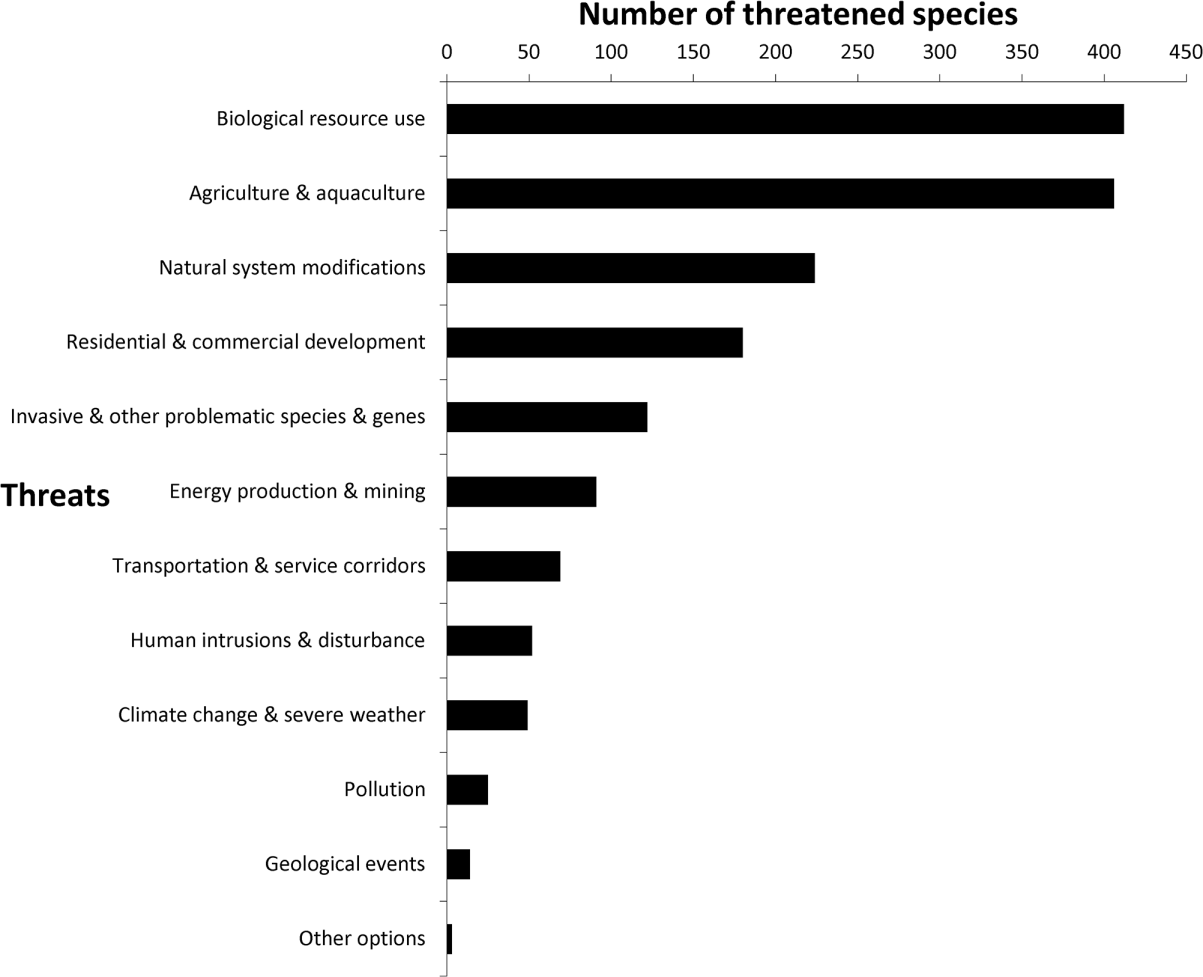
Threats to species from the combined SRLI for Plants sample of gymnosperms, monocots, legumes and pteridophytes, by number of plant species threatened. Individual species may be affected by more than one threat.

Most of the threatened species identified in this study are restricted to very small areas and are threatened by habitat conversion (see also [31]), with ‘Biological Resource Use’ and ‘Agriculture, Aquaculture’ together accounting for 50% of identified threats to plants (Fig 2). Within each of these broad classes, some key threats stand out. Arable farming affects 60% of threatened species, while livestock farming affects 47%, logging affects 38%, targeted harvesting affects 25% and fires (natural or man-made) also affect 25% of threatened species.

### SRLI value and geographic patterns

**Fig 3:**
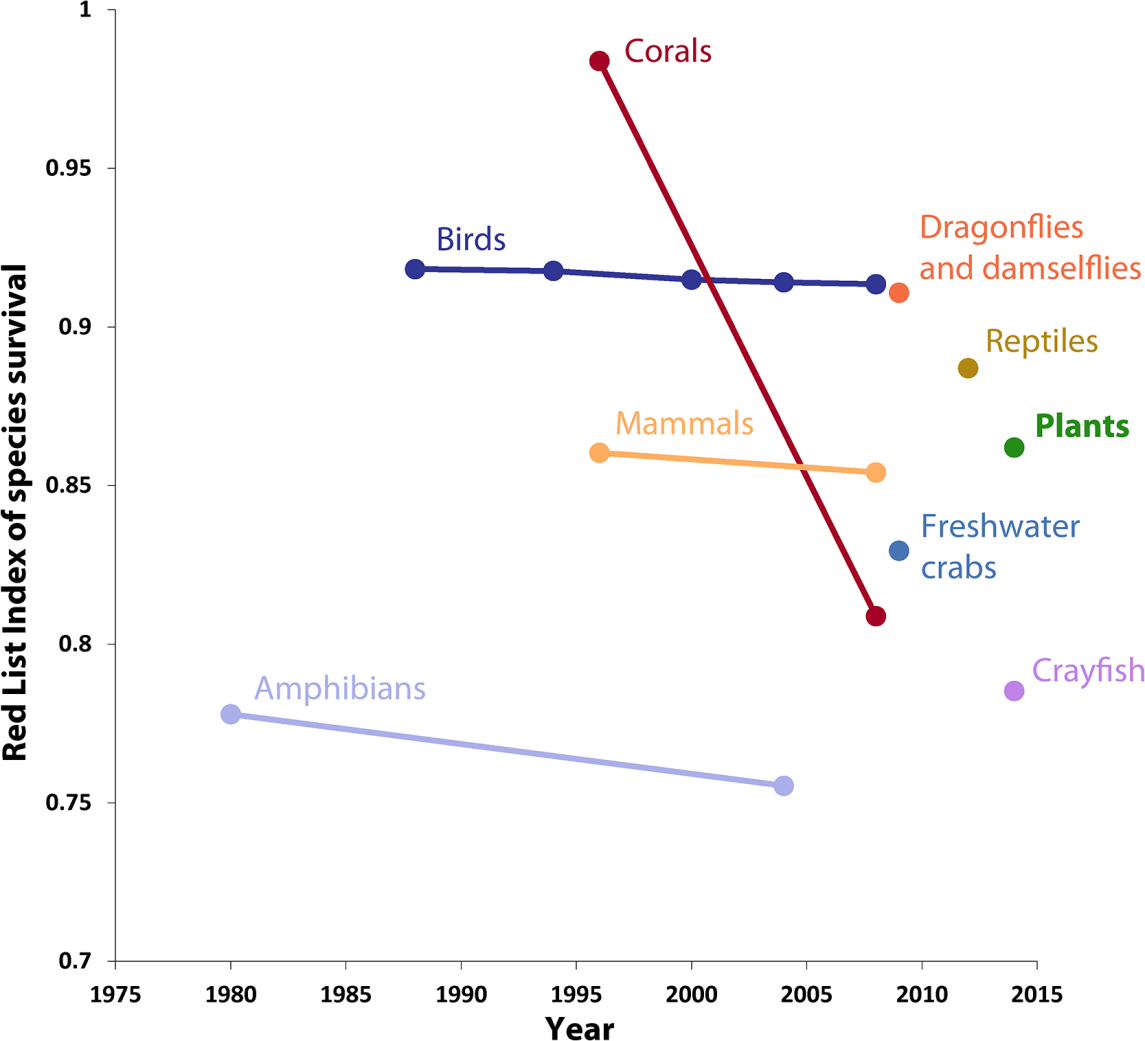
Red List Indices for birds, mammals, amphibians and corals (source: IUCN), with baseline values for crayfish [13], freshwater crabs [16], dragonflies & damselflies [17], reptiles [19] and plants (this study). Values for crayfish, freshwater crabs, dragonflies and damselflies, reptiles and plants are based on a sampled approach.

**Fig 4:**
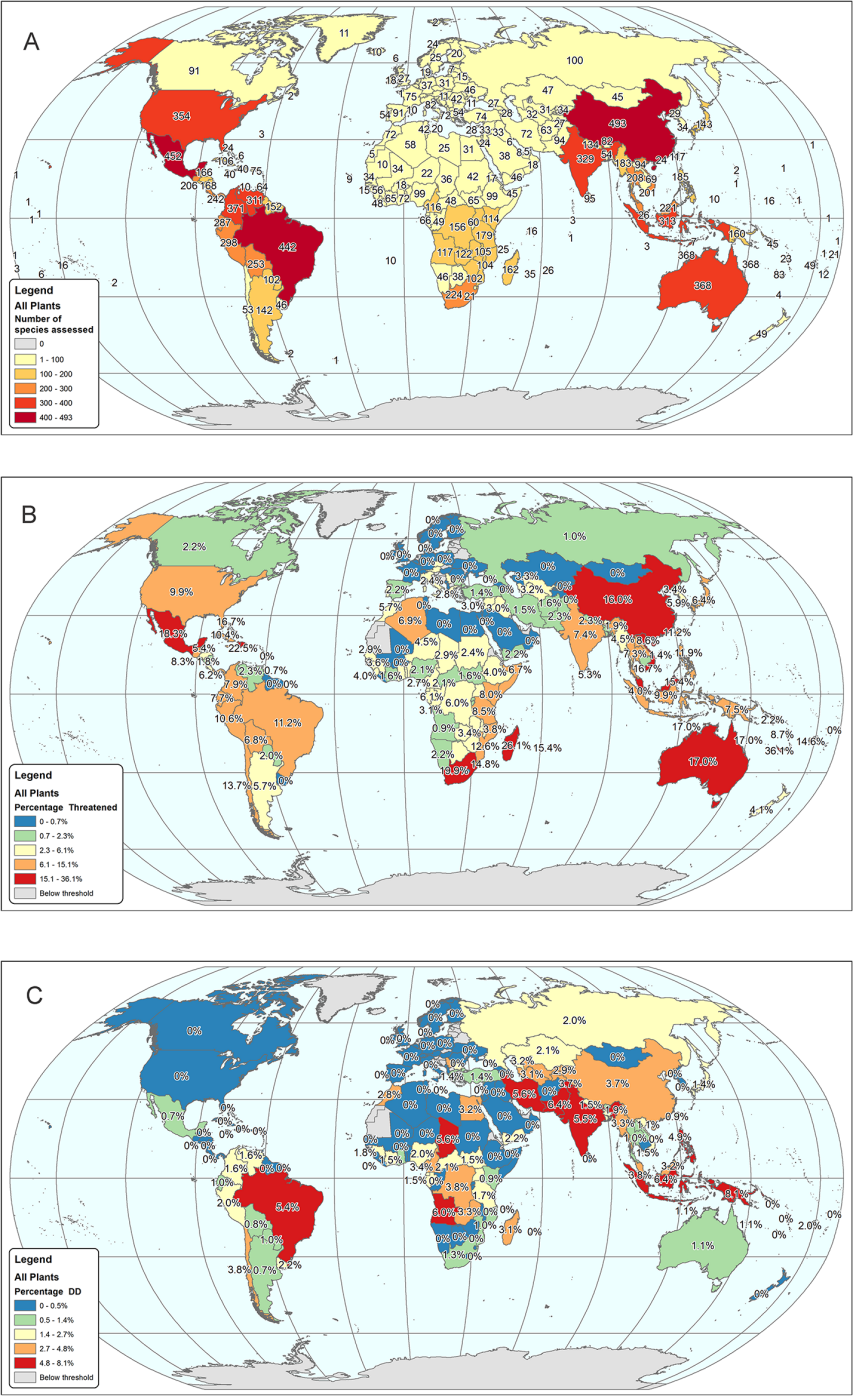
Global map of average extinction risk of species per country from the combined SRLI for Plants sample of gymnosperms, monocots, legumes and pteridophytes. A. Number of species assessed per country. B. Percentage of assessed species that are threatened per country. C. Percentage of assessed species that are Data Deficient per country.

**Fig 5:**
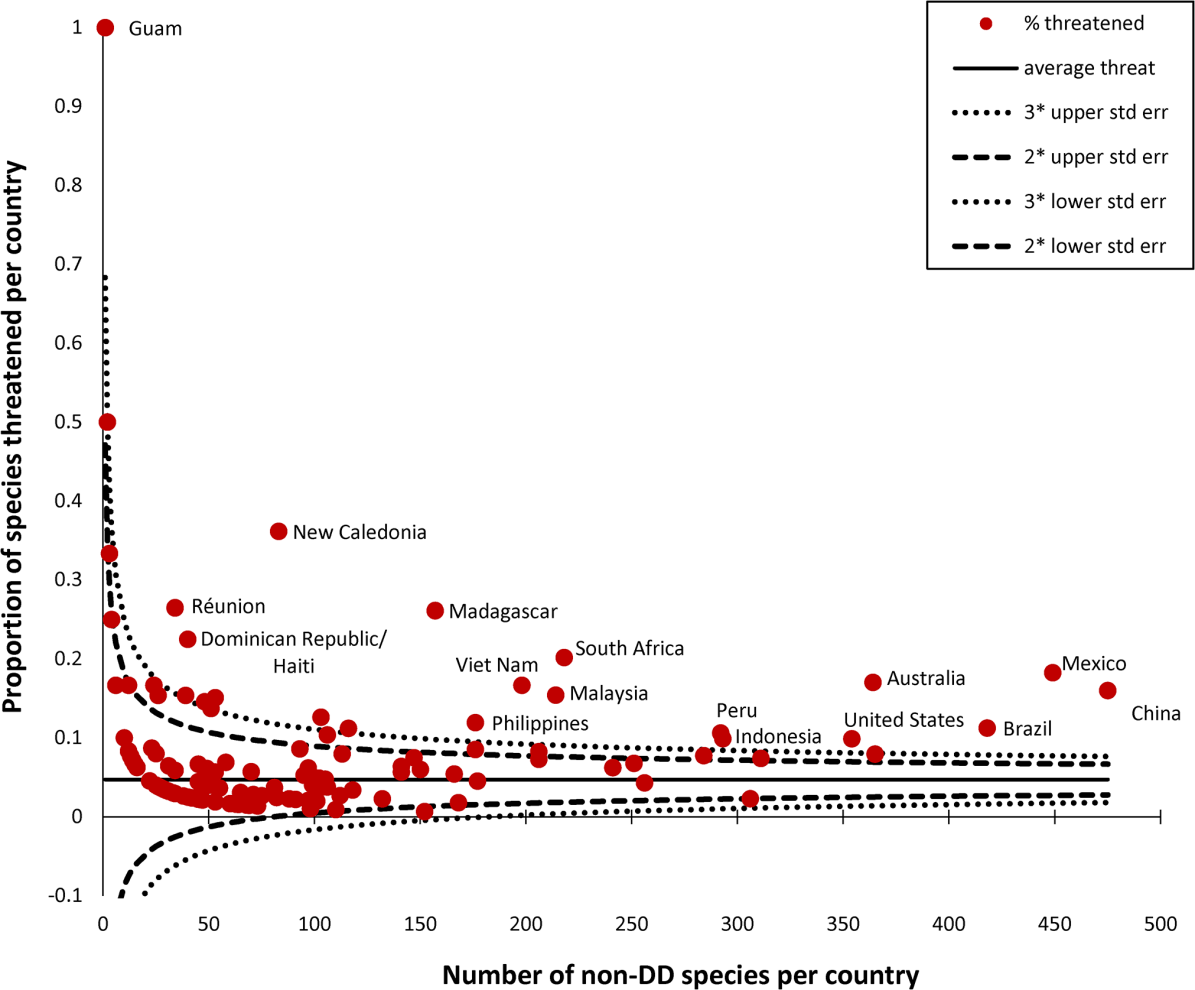
Funnel plot of degree of threat as a proportion of species assessed per country for the combined SRLI for Plants sample of gymnosperms, monocots, legumes and pteridophytes.

The baseline value of the SRLI for Plants is 0.86 (Fig 3), almost equal to that for mammals (0.85); compared with the other completely-assessed vertebrate groups, plants are less threatened than amphibians (0.74) but are more threatened than birds (0.92). Numbers of species assessed per country are presented in Fig 4A, proportions of species assessed as threatened per country are given in Fig 4B and gaps in our current knowledge of plant extinction risk given in Fig 4C. The full number of assessed species per country is shown in Fig 4A, but for Fig 4B and 4C data were only displayed for countries with 17 or more species assessed as non-Not Evaluated, as determined from simulations (S3 Fig). Particular gaps in knowledge are evident in Brazil, in Angola and Chad in Africa, and in southern Asia from Iran and through SE. Asia to Papua New Guinea (Fig 4C). A country-level funnel plot of the proportion of species that are threatened against number of species (Fig 5) also shows those countries with especially high proportions of threatened species, with several of the ‘megadiverse’ [54] countries (Madagascar, South Africa, Mexico, Brazil, Australia and China, and also Viet Nam and Malaysia, along with New Caledonia and Reúnion) having disproportionately high numbers of threatened species in this sample.

## Discussion

From our analysis of the SRLI for Plants, it is possible to say for the first time which plants from the sample are at greater risk of extinction, where and why. The sample of species assessed for this project is broadly representative of the world’s plants as a whole, and collectively there are sufficient species assessed for it to also be representative of some broad-scale geographical patterns. Previous work [14] determined an appropriate sample size for the SRLI on purely statistical grounds, and assessed the taxonomic and geographic coverage of samples retrospectively, whereas here the appropriate sample size has been determined independently of any single sample of species. By combining the results for each of the major plant groups together a robust and more detailed picture of the threats facing plant diversity emerges; these results are based on many thousands of assessments of individual species that in turn are based on hundreds of thousands of individual plant records.

### The threatened plants

Species from tropical regions are, in general, about twice as likely to be threatened as are species from temperate regions (21.22%–22.53% species threatened for tropical regions, 10.05%–12.2% species threatened in north temperate regions; Table 2); this may be partly attributable to temperate species having naturally larger ranges [55] and thus being less severely affected by localised threats. Other factors contributing to the marked contrast in proportions of threatened species between temperate and tropical regions may include the greater rate of land-use change occurring in the tropics, and the fact that in temperate areas such as Europe much of the change in land use and habitat cover has occurred a long time ago (i.e. more than 3 generations ago for most plant species) and native forest has been replaced by stable grassland or heathland communities, hence the species left have depleted populations but are no longer declining. In tropical regions there is typically much less detailed knowledge of the distribution of rare species, and also many more naturally rare species occur in the tropics across a greater diversity of habitats [56]. With increasing levels of habitat loss, the extinction of locally endemic species is highly likely.

### Non-threatened species

Almost two-thirds of plant species assessed for this study are classified as Least Concern. However, in many cases there has already been a reduction in the quantity or quality of natural habitat across the ranges of these species, although the range or population of these species have not yet declined sufficiently fast or to sufficiently low values to meet the thresholds set by IUCN for a threatened category, in some cases because the species is known to occur within effectively managed protected areas. In addition, some are naturally small-ranged, but are not currently undergoing range or population decline despite falling under the IUCN EOO threshold for Vulnerable. Conversely, as we have taken a randomly-selected sample representative of all plants more broadly, a few species in this study are aggressive colonisers with extremely widespread distributions, for example bracken (*Pteridium aquilinum*). The high proportion of ‘data insufficient’ ratings–either Not Evaluated (those that at first glance did not appear to have enough information on which to base an assessment) and those formally assessed as Data Deficient–in the sample of species tells a story in its own right: about as many new plant species are described each year as are published on the IUCN Red List, and the biodiversity of many parts of the world, especially in inaccessible regions of the tropics, still remains largely unexplored. Many species have received no further attention since they were first described many decades ago, and much of the baseline research on what plant species occur where in the world is still in progress.

### Legumes as proxies for broader angiosperm patterns

The SRLI for Plants is calculated giving equal weight to each taxonomic group included in the index, following Baillie et al. [14]. However, if the value of the SRLI plants were to be re-calculated (weighted) to reflect the total number of species for each taxonomic group of plants, in their current proportions between IUCN Red List Categories (i.e. the ‘best’ estimate of proportions threatened, across all species), still using the legumes as a proxy for all angiosperms other than monocots, then the SRLI value would increase to 0.91, or 1 in 7 plant species (results not shown). Weighting each group equally emphasizes an equivalent degree of knowledge of species extinction risk, whereas weighting each group in proportion to its number of species effectively gives greater weight to groups that are less well studied and less valued by the public [14], which is arguably less comprehensible and less useful to policy and decision makers. Whether legumes are a good proxy for the extinction risk of other non-monocot angiosperms can only be established with certainty when a sample including other families has been assessed. However, additional studies further suggest that legume diversity may serve as a proxy for total plant diversity in biogeographical and ecological analyses [57–61]. Resources permitting, a broader sample of non-monocot angiosperms will be included in the next round of assessments for the SRLI for Plants.

### Goodhart’s Law

The value of the IUCN Sampled Red List Index for Plants has been estimated from robust assessments of several thousand species using available information, yet this represents only a very small proportion of the total number of plants. What ensures that the value of the SRLI for Plants truly represents the status of the rest of plant diversity? Targeted conservation actions aimed only at SRLI species could disproportionately influence the value of the Index, suggesting a healthier picture for global plant extinction risk than is the case for non-SRLI plant species–the vast majority of species. The imposition of targets measuring performance often precipitates changes in the behaviour of those responsible for the performance of the system–a phenomenon described by Goodhart’s Law [62]. What is to stop the small selection of plant species becoming the target of specific conservation actions designed merely to improve the status of the species sampled, and hence the reading of the Index, with no commensurate improvement in the status of other species?

While acknowledging that action targeted disproportionately on species in the SRLI sample may risk incorrect inferences from future readings of the Index, we consider that the absolute rather than relative number of plant species selected and assessed to date, the range of taxonomic groups and the global geographical and ecological coverage achieved all mitigate this risk. Moreover, the current (non-Sampled) Red List Index includes assessments for over 20,000 and species of birds, mammals, amphibians and corals, and the SRLI as a whole, once samples are re-assessed and incorporated into a combined index, will include not only plants but assessments of tens of thousands of species representing many more groups of organisms [14,16–19] It is hard to see how a positive change in the Index as a whole would not genuinely represent a positive improvement in the status of global biodiversity. Furthermore, the assessments for plant species are often based on use of criterion B, as discussed above, which requires that two out of three subcriteria need to be met, one of which relates to a decline in size and structure of species’ range or quality of habitat. This criterion (B1 and/or B2) has been used for 59% of threatened plant species assessed for the SRLI for Plants [29]. For species to be assessed as threatened under criterion B, two out of three subcriteria also need to be fulfilled; in the majority of cases this is a severely fragmented distribution or small number of locations (subcriterion a), together with a recent and ongoing decline in either the range of the species or in the quality or the extent of its habitat (subcriterion b iii). This means that for the majority of threatened SRLI plant species, a positive change in their IUCN Red List status may also mean an improved situation for non-SRLI species found within the range or the same habitat as those SRLI species receiving careful attention.

### Plants in comparison with other organisms

The SRLI is scaled between 1 and 0, where a value of 1 would indicate that every species included in the Index was Least Concern, whereas a value of zero would indicate that every species in the index had gone extinct [41]. The value for all plant groups included in the SRLI for Plants [3990 species] is 0.86, showing that overall, plants have a comparable level of extinction risk as do mammals [0.85], are much more threatened than are birds [0.91], but are much less threatened than are amphibians [0.76] (c.f. [52]). For other taxonomic groups from which samples have been assessed, SRLI values range from 0.91 (dragonflies and damselflies) to 0.79 (freshwater crayfish). Warm water reef-building corals show the steepest decline in extinction risk of any completely assessed group, largely due to the impacts of climate change on ocean acidification, ocean warming and reef formation, and to local habitat destruction caused by over-fishing of reef-dwelling fish [12]. Countries with a high proportion of threatened plant species are those ‘megadiverse’ countries [54] whose floras constitute a confluence of many different floristic elements [63]. These same countries are also areas of high vertebrate diversity and threat [64–65]. Therefore, the future of plant diversity depends on the conservation actions taken in the next few years in combination with efforts to conserve the world’s animal species [56,66].

### Life depends on plants–safeguarding our future

The results from the SRLI for Plants graphically show the impact of humans on the state and fate of plant diversity. Most threatened species are found in the tropics, where the greatest diversity of plants is [39], with 21–23% threatened compared to 10–12% threatened in temperate regions (Table 2). Oceanic islands have fewer species but these are often found nowhere else, and are more likely to be threatened, especially by the introduction of invasive species [67]. Threatened species identified in this study have mostly (59%) been assessed under Criterion B, and are predominantly narrow endemics threatened by habitat conversion, or species naturally confined to very restricted areas [29] ‘such that they are prone to the effects of human activities or stochastic events within a very short time period in an uncertain future’ [42] and are thus liable to become Critically Endangered or Extinct in a very short time period.

The habitat with the greatest number of threatened species is tropical rain forest, where 79% of threatened species are found. Changes in habitat cover can be clearly seen from satellite images, and many localities where specimens have been collected within recent years would now be unrecognisable. The overwhelming threat affecting the future of plant species is the continued destruction of natural habitats and their ongoing degradation or conversion to agriculture. Areas least suitable for conversion to agriculture, such as wetlands and deserts, contain the smallest proportions of threatened plant species, with 3% and less than 1% of threatened species, respectively. Land use change from natural to man-made habitats is the major driver of changes in the provision of ecosystem services, and the Millennium Ecosystem Assessment has forecast that 10–20% of current grassland and forested land may be lost between now and 2050, mainly due to the further expansion of agriculture [68]. This fragments the ranges of already-threatened species further, isolating or eliminating populations and preventing their natural dispersal and ability to reproduce. Plants are the primary producers for all terrestrial and almost all aquatic ecosystems. They have roles of fundamental importance in providing food, shelter and building materials for humans as well as for many animal species, for regulating climate, providing supporting ecosystem services such as soil formation, preventing soil erosion and desertification, filtering fresh water, nutrient cycling, photosynthesis and for structuring habitats. More than one in five plant species assessed is already threatened with extinction, even without considering the threats associated with a changing climate. A trait-based assessment of the vulnerability of species of other taxonomic groups to climate change has shown large numbers of species that are not currently threatened but which are highly vulnerable to climate change impacts (potentially as many as 41% of bird species, 29% of amphibian species and 22% of reef-forming corals [69]). If the same patterns hold true for plants, the number of plant species of conservation concern is likely to rise sharply in the coming decades. The current rate of loss of tropical forest accounts for 20% of global carbon emissions, which means that taking the necessary steps to reduce biodiversity loss will also make a significant contribution to mitigating the impact of climate change. This would be a win-win situation for our own species and countless thousands of others.

### Phase II of the SRLI for Plants: re-assessing the species

All assessments carried out for this project are underpinned by accurate and reliable information on where and when a species has been collected, with an auditable data trail to the source data, and are validated by the opinion of taxonomic or regional experts and double-checked by the IUCN Red List Unit. However, most are based on knowledge of the species’ range and habitats and information about the current vegetation cover within that area: in common with almost all plant species, few have been recently surveyed in their natural habitat. Doing so would greatly enhance the existing assessments by enabling the use of a wider range of the IUCN Red List Criteria and improving the rigour of ratings that are generally based on a remote analysis of species ranges from recent and historical herbarium specimen data. Using the associated specimen data for each assessment, hotspots of threatened plant species diversity can be identified at higher spatial resolution, and targeted field expeditions to survey these threatened species in the wild will concentrate in these areas, providing a focussed framework for conservation actions and ensuring that resources are used effectively and where they are most needed [29,70–73].

In order for changes in the value of the IUCN Red List Index to be seen, changes in the status of each species need to be detected [8–9], and so these species need to be regularly surveyed. The aim is for each species to be re-assessed every five years, and the Index re-calculated; species which have undergone a genuine change in their extinction risk then drive the change in the value of the Index. If an international network of scientists and relevant institutions such as botanic gardens and natural history museums can be mobilised, and monitoring schemes established or expanded at national scales [26] then the baseline presented here can be built on to accurately monitor future trends in the status of plant diversity worldwide. The use of web platforms to harvest and collate photographic records and population survey data (e.g. www.inaturalist.org), the analysis of long-term Earth Observation data [74] to better understand declines in populations and the harnessing of data from citizen science-type initiatives [75] are all likely to underpin the next phase of this work [29].

## Conclusion

This initial phase of the SRLI for Plants marks the first step towards monitoring global extinction risk in plant species. It provides the baseline against which future changes can be tracked–and it shows clearly that urgent action is needed if we are to avoid losing one in five plant species. Subsequent assessments of this sample will provide a trend in plant extinction risk, indicating whether extinction of threatened species has been prevented and whether the status of those species most in decline has been improved, directly responding to Aichi Target 12. In addition, being the first representative assessment of extinction risk of plants it also provides the first direct response to a milestone of the second target of the Global Strategy for Plant Conservation (GSPC). We hope the methods and techniques uses in this study, as well as the headline results, will act as a stimulus to further plant extinction risk assessment activity and help galvanise and mobilise the international network of local botanists and botanic gardens to establish the SRLI for Plants as a broad-based monitoring scheme. The world cannot afford to lose a quarter of its plant species; we must all work together to conserve what we have.

## Supporting Information

S1 Fig Diversity statistics based on species numbers of monocots distributed across families. Minimum and maximum estimates obtained across different sample sizes are shown for Shannon-Weiner (squares) and Simpson (diamonds) diversity indices; lines within the bounds represent diversity estimates for monocots as a whole.
(DOC)

S2 Fig Diversity statistics based on species numbers of monocots distributed across geographic regions. Minimum and maximum estimates obtained across different sample sizes are shown for Shannon-Weiner (squares) and Simpson (diamonds) diversity indices; lines within the bounds represent diversity estimates for monocots as a whole.
(DOC)

S3 Fig Plot of standard error against number of species sampled for 5,000 random simulations. The threshold value used for showing countries on the maps in Fig 4 (17 species) is highlighted in red.
(DOCX)
