# Supplementary figures and images for: Green Plants in the Red: A Baseline Global Assessment for the IUCN Sampled Red List Index for Plants
Source: PLoS One. 2015 Aug 7;10(8):e0135152. doi: 10.1371/journal.pone.0135152 (PMC4529080; doi:10.1371/journal.pone.0135152)

**Figure S1** Diversity Simulation part 1 – Families


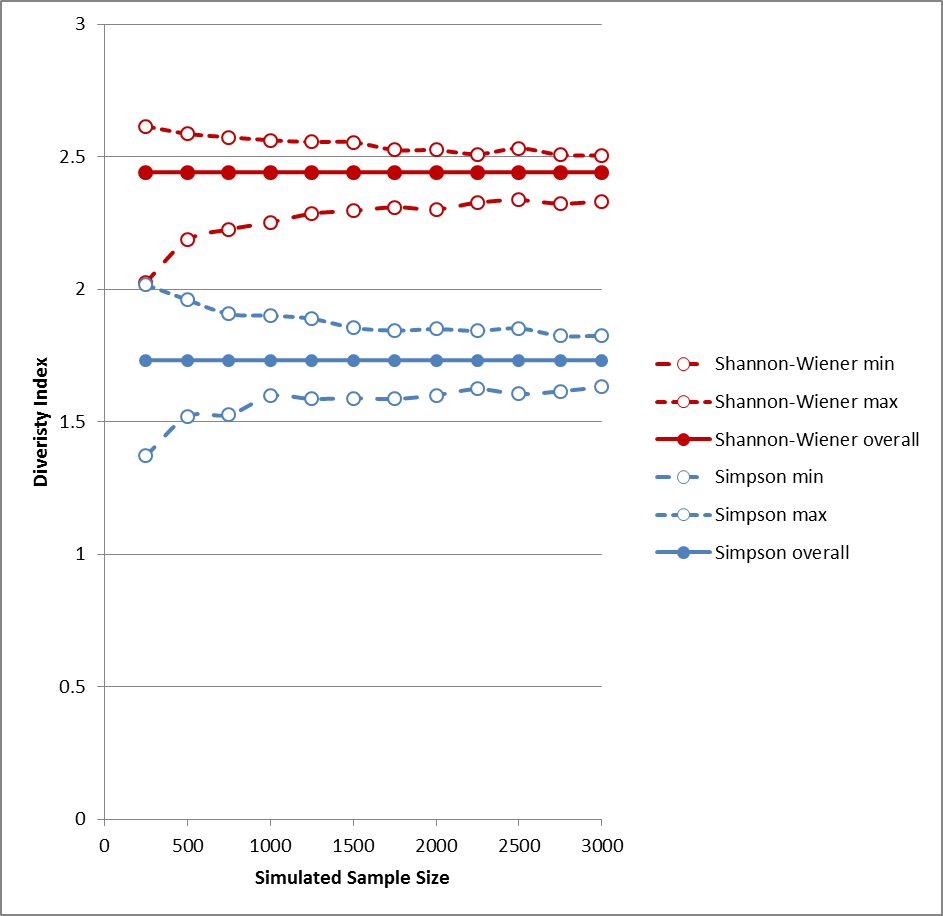

Supplement: S1 Fig — Minimum and maximum estimates obtained across different sample sizes are shown for Shannon-Weiner (squares) and Simpson (diamonds) diversity indices; lines within the bounds represent diversity estimates for monocots as a whole. (DOC) [file pone.0135152.s001.doc]

**Figure S2** Diversity Simulation part 1 – Geographic Regions


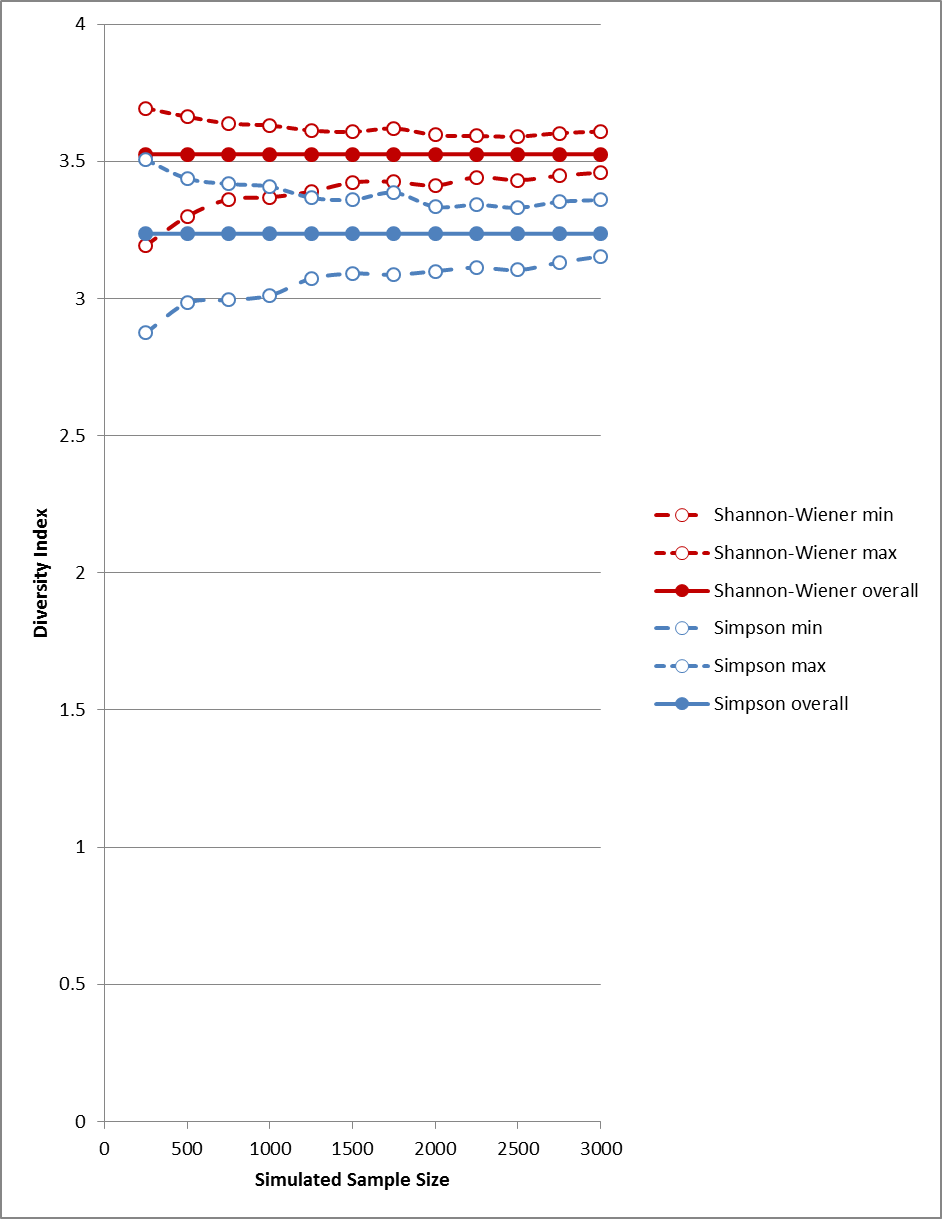

Supplement: S2 Fig — Minimum and maximum estimates obtained across different sample sizes are shown for Shannon-Weiner (squares) and Simpson (diamonds) diversity indices; lines within the bounds represent diversity estimates for monocots as a whole. (DOC) [file pone.0135152.s002.doc]
