# Supplementary material for: Green Plants in the Red: A Baseline Global Assessment for the IUCN Sampled Red List Index for Plants
Source: PLoS One. 2015 Aug 7;10(8):e0135152. doi: 10.1371/journal.pone.0135152 (PMC4529080; doi:10.1371/journal.pone.0135152)
Supplement: S3 Fig — The threshold value used for showing countries on the maps in Fig 4 (17 species) is highlighted in red. (DOCX) [file pone.0135152.s003.docx]

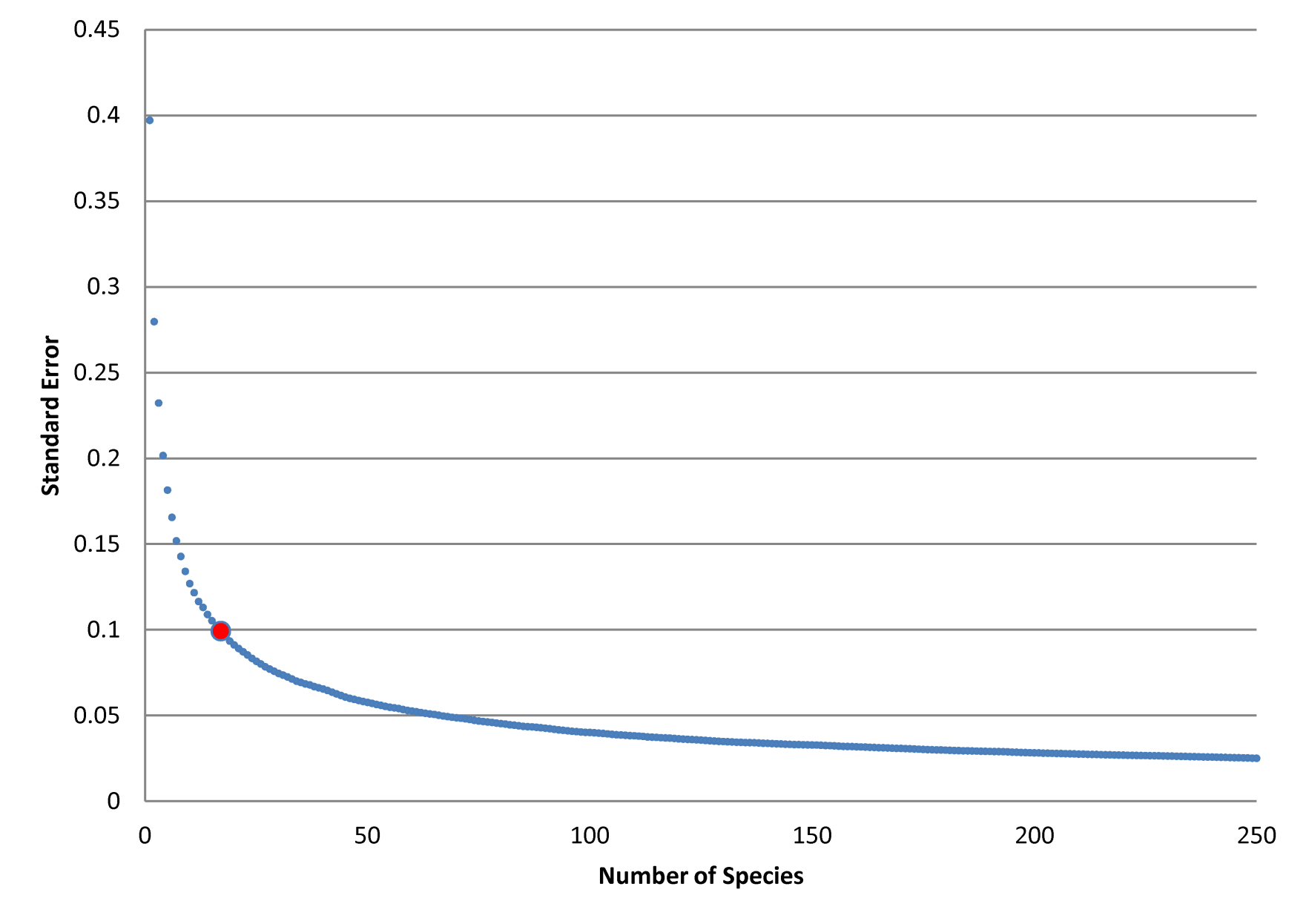


**Supplementary Figure S3 Plot of standard error against number of species sampled for 5,000 random simulations. The threshold value used for showing countries on the maps in Figure 4 (17 species) is highlighted in red.**
